# Identification of a QTL conferring resistance to the Subtropical Race 4 of *Fusarium oxysporum* f. sp. *cubense* in Calcutta 4 (*Musa acuminata* ssp. *burmannica*)

**DOI:** 10.1093/hr/uhag001

**Published:** 2026-01-09

**Authors:** Andrew Chen, Guillaume Martin, Altus Viljoen, Jiaman Sun, Emily Rames, Nabila Yahiaoui, Angelique D'hont, Brett J Ferguson, Rony Swennen, Robert J Henry, Rajeev K Varshney, Elizabeth A B Aitken

**Affiliations:** School of Agriculture and Food Sustainability, The University of Queensland, Brisbane, QLD 4072, Australia; CIRAD, UMR AGAP Institut, F-34398 Montpellier, France; UMR AGAP Institut, Université Montpellier, CIRAD, INRAE, Institut Agro, F-34398 Montpellier, France; Department of Plant Pathology, Stellenbosch University, Stellenbosch 7600, South Africa; School of Life Science, Jiaying University, Meizhou 514015, China; Department of Primary Industries, Maroochy Research Facility, Nambour, QLD 4560, Australia; CIRAD, UMR AGAP Institut, F-34398 Montpellier, France; UMR AGAP Institut, Université Montpellier, CIRAD, INRAE, Institut Agro, F-34398 Montpellier, France; CIRAD, UMR AGAP Institut, F-34398 Montpellier, France; UMR AGAP Institut, Université Montpellier, CIRAD, INRAE, Institut Agro, F-34398 Montpellier, France; Integrative Legume Research Group, School of Agriculture and Food Sustainability, The University of Queensland, Brisbane, QLD 4072, Australia; International Institute of Tropical Agriculture, Banana Breeding, PO Box 7878, Kampala, Uganda; Department of Biosystems, KU Leuven University, Willem De Croylaan 42, bus 2455, 3001 Leuven, Belgium; Queensland Alliance for Agriculture and Food Innovation, University of Queensland, Brisbane, QLD 4072, Australia; ARC Centre for Plant Success in Nature and Agriculture, University of Queensland, Brisbane, QLD 4072, Australia; Centre for Crop and Food Innovation, WA State Biotechnology Centre, Food Futures Institute, Murdoch University, Murdoch, WA 6150, Australia; School of Agriculture and Food Sustainability, The University of Queensland, Brisbane, QLD 4072, Australia

Dear Editor,

Fusarium wilt of banana (FWB), caused by *Fusarium oxysporum* f. sp. *cubense* (*Foc*), continues to be one of the most devastating diseases of bananas worldwide. The pathogen is highly adaptive, and composed of distinct evolutionary lineages, and three races with pathogenicity to specific banana cultivars. *Foc* Race 1 decimated the Gros Michel (AAA genome) based banana industry in the 1950s and was replaced with the Cavendish banana, which is resistant to *Foc* Race 1. Since then, Cavendish has dominated the global market but is susceptible to infection by *Foc* Race 4, which includes Subtropical Race 4 (STR4) and Tropical Race 4 (TR4). Whereas TR4 infects Cavendish in all environments where banana is grown, STR4 is a lineage closely related to TR4 that causes disease in subtropical regions with cooler climates. Since the 1990s, global banana production has succumbed to TR4, after it was first detected in the Asia-Pacific region. The fungus has since spread to many banana growing regions around the world. In Australia, TR4 remains confined to the Tully Valley in northern Queensland since its first detection in 2015, yet the long-term sustainability of the country's banana industry is under ongoing threat. With no effective chemical control available, resistance breeding is widely considered the most promising management strategy. Wild diploid accessions of *Musa acuminata* are key sources of resistance to *Foc* Race 4. So far, quantitative trait locus (QTL) for STR4 resistance has been identified in *M. acuminata* ssp. *malaccensis* [1]. Here we report the first genetic dissection of resistance from *M. acuminata* ssp. *burmannica*, another valuable diploid group in banana improvement programs.

In this study, a bulk segregant and sequencing approach was used to detect chromosome regions conferring STR4 resistance in a *M. acuminata* ssp. *burmannica* derived F_2_ population (Fig. 1A). For this, pollinations were conducted to obtain an intercross population between ‘Ma848’ (*M. acuminata* ssp. *malaccensis*, STR4 susceptible, female parent) and ‘Calcutta 4’ (*M. acuminata* ssp. *burmannica*, ITC0249, STR4 resistant, male parent), and populations derived from the selfing of each parent (Fig. 1B). Chromosome ancestry painting [2] using ancestry informative alleles revealed that ‘Calcutta 4’ has a genome-wide homogenous contribution of the *M. acuminata* ssp. *burmannica* origin (Fig. 1C), whereas ‘Ma848’ appeared predominantly derived from *M. acuminata* ssp. *malaccensis* origin, but with introgressed segments originating from other subspecies (Fig. 1D).

**Figure 1:**
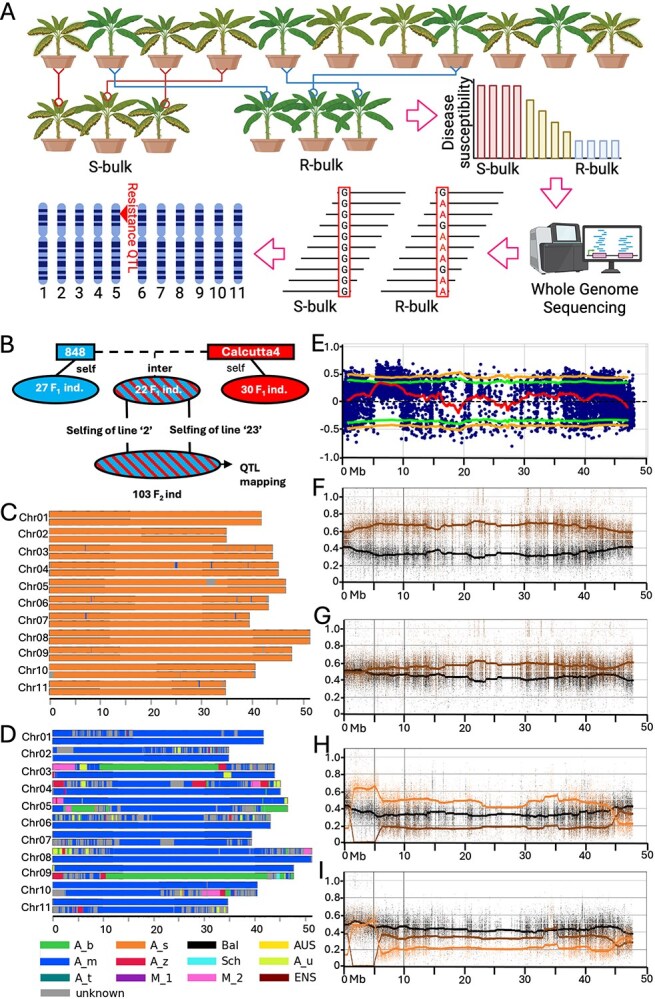
Identification of a QTL conferring resistance to *Fusarium oxysporum* f. sp. *cubense* Subtropical Race 4 in *Musa acuminata* ssp. *burmannica*. (A) A graphical illustration of the bulk-segregant and whole-genome sequencing approach used in this study to identify the resistance QTL. (B) Pedigree of the crosses made in this study. Red colour annotates the source of resistance. Blue colour annotates a lack of resistance. Alternate red/blue pattern annotate the segregation of STR4 resistance. ‘ind.’ abbreviates individuals; ‘self’ abbreviates self-cross; ‘inter’ abbreviates inter-cross. (C, D) Genome ancestry mosaic painting for (C) ‘Calcutta 4’ and (D) ‘Ma848’. Ancestral group contributions are presented along the 11 *Musa acuminata* ssp. *malaccensis* reference chromosomes (DH-Pahang v4) by different colours. A_b, *Musa acuminata* ssp. *banksii*; A_s, *Musa schizocarpa*; Bal, *Musa acuminata* ssp. *balbisiana*; AUS, *Australimusa*; A_m, *Musa acuminata* ssp. *malaccensis*; A_z, *Musa acuminata* ssp. *zebrina*; Sch, *Musa schizocarpa*; A_u, *Musa acuminata* ssp. *sumatrana*; A_t, *Musa acuminata* ssp. *truncata*; M_1, *Musa acuminata* ssp. *halabanensis*; M_2, unknown 2; ENS, *Musa ensete*; unknown, SNPs not assigned to any of the known genetic groups. The scale is indicated in Mb at the bottom of the figure. The representation is based on non-phased genotyping data. (E) Changes in SNP-index were displayed across chromosome 3 of ‘AF-Calcutta 4’ in a window size of 2 Mb and a fixed step size of 100 Kb. Red, green and yellow lines indicate ΔSNP-indices, a confidence interval at 95% and 99%, respectively. Only SNPs with SNP-index values greater than 0.5 were plotted. (F, G) Ancestry painting of the resistant bulk (F) and susceptible bulk (G) using SNPs assigned to each of the grandparents, ‘Calcutta 4’ (brown) and ‘Ma848’ (black). (H, I) The grandparent haplotype painting of the resistant bulk (H) and susceptible bulk (I) using SNPs assigned to ‘Calcutta 4’ haplotype 1 (brown) and 2 (orange), and ‘Ma848’ (black). *Musa acuminata* ssp. *burmannica* ‘AF-Calcutta 4’ v2 was used as a reference.

To confirm the segregation of resistance at the F_1_ generation, 22 F_1_ lines (‘Ma848 × Calcutta 4’, *n* = 1–4 clone per line), grown under a controlled environment, were challenged with a spore suspension extracted from *Foc* strains carrying Vegetative Compatibility Group or VCG 0120 (Queensland Plant Pathology Herbarium or BRIP: 63488, 43 781, and 42 331, all isolated from Cavendish hosts), and then scored for symptoms at 10 to 12 weeks post inoculation [3]. Half of these lines (50%) were considered resistant, having a mean rhizome discolouration score between 0% and 20%. This suggests that ‘Calcutta 4’ is heterozygous for STR4 resistance. This was further supported by the segregation of resistance in 30 self-derived ‘Calcutta 4’ F_1_ lines at a ratio of 3.3:1 (R:S). The resistant ‘Ma848 × Calcutta 4’ F_1_ lines ‘2’ and ‘23’ were selected and selfed to establish an F_2_ population. A total of 103 lines were recovered after embryo culture and 1–4 clones per line were challenged with STR4. Disease scoring showed that over 50% of lines had ≥20% rhizome discolouration.

Based on the rhizome discolouration index, individual DNA of the 30 most resistant and susceptible F_2_ lines were combined into an R-bulk and an S-bulk, respectively. Both bulks and the ‘Calcutta 4’ parent were sequenced using Illumina Novaseq 6000. A QTLseq pipeline [4] was then used for raw data processing and SNP identification on the ‘AF-Calcutta 4’ assembly [5], and for calculating changes in SNP indices to identify QTLs associated with resistance. A significant QTL was detected at a 95% confidence interval in the 5- to 10-Mb region on the short arm of chromosome 5 (Fig. 1E). No other QTLs were detected at the 95% or 99% confidence level.

The contributions of the population’s grandparents to the bulks were investigated along chromosomes, first by using specific alleles for each grandparent, and then at haplotype level for ‘Calcutta 4’. For this, variant calling was performed on ‘Calcutta 4’ and ‘Ma848,’ and a parent–child trio from a previous study consisting of the F1–061 individual (ERS14379603) and its parents ‘Pahang’ (PT-BA-00267, ERS14379600) and ‘Calcutta 4’ (ERS14379599) [2]. The alleles specific to ‘Ma848’ or ‘Calcutta 4’ were identified and their ratios in the R- and S-bulks were displayed along chromosomes. This showed that in the QTL region, the ‘Calcutta 4’ contribution was higher than the ‘Ma848’ contribution in the R-bulk (Fig. 1F and G). In contrast, the coverage of both appeared relatively even across this region in the S-bulk (Fig. 1G). Using the parent–child trio data, phasing (without taking into account recombination events) allowed access to the ‘Calcutta 4’ haplotypes. The alleles specific to ‘Ma848’ and to each ‘Calcutta 4’ haplotype were then determined and their ratios analysed in the bulks. In the QTL region, one can observe a contrasted genomic contribution of ‘Calcutta 4’ haplotypes with the R-bulk enriched in haplotype 2 and the S-bulk enriched in haplotype 1 (Fig. 1H and I), indicating that ‘Calcutta 4’ haplotype 2 is associated with the resistance QTL. A heterozygous interstitial inversion from bp position 6 148 099 to 7 151 701, supported by evidence of Illumina split-read alignments at both breakpoints, was detected in the QTL region of ‘Calcutta 4’ when it was aligned to a corresponding region in *M. acuminata* ssp. *malaccensis*, Cavendish, Pisang Madu H1, *M. acuminata* ssp. *banksii*, *M. acuminata* ssp. *zebrina*, *M. balbisiana*, and *M. schizocarpa*. This tends to suggest that this inversion is exclusive to this genotype.

Using SnpEff and SnpSift programs (https://pcingola.github.io/SnpEff/), loss-of-function and missense variants in the 5- to 10-Mb candidate region on chromosome 5 were detected and then filtered to obtain those having a significant ΔSNP-index at a 95% confidence level. Two loss-of-function (LOF) and 36 missense mutations were subsequently identified. The LOF mutations included a frame-shift variant in the serine/threonine-protein kinase (Mabur_05g008220.1) at 6.3 Mb and a splice acceptor site mutation in a gene encoding a conserved protein of unknown function (Mabur_05g010680.1) at 8.0 Mb. Both mutations were detected in the S-bulk relative to the R-bulk and the ‘AF-Calcutta 4’ assembly.

While genomic resources for banana research have expanded greatly in recent years, the underlying genetic methods for detecting QTLs for crop improvement have not progressed as much. The lack of progress in identifying candidate genes for *Foc* resistance is partly due to challenges associated with phenotyping banana and difficulty of developing large, segregating populations for these traits. In this study, we have identified, for the first time, a QTL responsible for resistance to STR4 in *Musa acuminata* ssp. *burmannica*. This resistance locus can now be further analysed to identify candidate genes for subsequent functional validation *in planta*. Additionally, molecular markers can be developed to track the inheritance of resistance alleles in banana breeding programs. Overall, this work represents a significant step toward combating and mitigating the TR4 pandemic, which continues to devastate banana plantations worldwide.

## Data Availability

The original contributions presented in the study are included in the article. The Illumina raw sequence data for ‘Calcutta 4’ and ‘Ma848’ are available from the NCBI (National Center for Biotechnology Information) BioProject database under accession number PRJNA1333582. Bulk sequence data are not publicly available due to confidentiality related to gene discovery. Requests for access or further information should be directed to the corresponding authors.
